# Social and Cognitive Skills (SCOPE)—a generic model for multi-professional work and education in healthcare

**DOI:** 10.1186/s41077-024-00302-6

**Published:** 2024-07-02

**Authors:** Peter Dieckmann, Birgitte Bruun, Sofie Mundt, Ragnhild Holgaard, Doris Østergaard

**Affiliations:** 1 Copenhagen Academy for Medical Education and Simulation (CAMES), Center for Human Ressources and Education, Capital Region of Denmark, Herlev Hospital, Borgmester Ib Juuls Vej, 1b, DK-2730 Herlev, Denmark; 2 Department of Quality and Health Technology, Faculty of Health Sciences, University in Stavanger, Rennebergstien 30, N-4021 Stavanger, Norway; 3 Department of Public Health, Copenhagen University, Øster Farimagsgade 5, DK-1353 Copenhagen, Denmark; 4 The Regional Secretariat for Postgraduate Medical Education East, Center for Human Ressources and Education, Gentofte Hospitalsvej 10B, Hellerup, Capital Region of Denmark 2900 Denmark; 5 Department of Clinical Medicine, University of Copenhagen, Blegdamsvej 3B, DK-2200 Copenhagen, Denmark

**Keywords:** Cognitive and social skills, Debriefing, Simulation

## Abstract

In this article, we present a generic model for social and cognitive skills that can be used in work and (simulation-based) education in healthcare. We combined existing non-technical skills tools into a tool that we call SCOPE. SCOPE is a model that comprises the three social categories of “teamwork”, “leading”, and “task management” as well as the two cognitive categories of “situation awareness” and “decision making”. Each category comprises between three and six elements. We formulated guiding questions for each category in an attempt to emphasize its core meaning. We developed a dynamic graphical representation of the categories that emphasize the constant changes in the relative importance of the categories over the course of a clinical or educational situation. Anecdotal evidence supports the value of the model for aligning language around social and cognitive skills across specialties and professions.

**Supplementary Information:**

The online version contains supplementary material available at 10.1186/s41077-024-00302-6.

## Introduction

The day had taken a lot of preparation and required a significant commitment from those delivering and participating in the event. Everyone was quite excited about this inter-professional development day. It was designed for personnel in the operating theatres of a large acute care hospital. There was obvious institutional buy-in. Except for those taking care of the most urgent cases, all were present: anaesthesiologists, scrub nurses, surgeons, and anesthesia nurses. Each of the professional groups—proud of their healthcare expertise, as well as their focus on patient safety and “human factors issues”—had their own tool to describe “non-technical skills”. We had placed all those tools as posters around the debriefing room. We finished the first simulation scenario. Technically it went off without a hitch. The room was buzzing when we got back to the debriefing room.

Then, the trouble started….

People were talking about teamwork but using different language. One debriefer asked a question to an anesthetist that just did not land—she clearly was not sure what the debriefer was talking about. You could see people in the room scanning the posters, trying to find their familiar framework. One scrub nurse asked: “What do you mean with leadership here? It is not in our tool?”. “Ah”, an anesthesia nurse supplemented, “what you discuss is in N-ANTS, but under task management, not leadership. Hm? Which one is right?”. The facilitators looked at each other, took a deep breath, and began to try to sort things, gesticulating towards different corners of the room at different posters with different tools.

The discussion of non-technical skills (NTS), comprising decision-making, situation awareness, teamwork, task-management, and leadership is important for patient safety, the quality of care, and the well-being of healthcare professionals [1–3]. Different professions and disciplines have therefore developed taxonomies that should guide the design and conduct of simulation sessions and debriefing sessions [4–10]. The ones that we discuss here all have several overarching categories, more detailed elements, and numerous positive and negative behavioral markers. The individual tools were tailored for specific professions in specific disciplines and therefore look very similar but are different on a detailed level. Table 1 provides an overview of the categories of the four tools used in Denmark. Notably, NTS tools typically do not include communication as a category or element, as they are thought to tie all the different categories and elements together (the Danish tools for surgeons [4] and scrub nurses [5] are exceptions here). Typically, these tools are used to discuss (and sometimes assess) non-technical skills for learning and/or research purposes.

**Table 1:** Overview of the categories in the Danish non-technical skills tools. Note that we translated the Danish words back to English for the purpose of this publication

NOTSSdk [4]	ANTSdk [10]	N-ANTS [6]	SPLINTSdk [5]
Situation awareness	Situation awareness	Situation awareness	Situation awareness
Decision making	Decision making	Decision making	
Communication and teamwork	Teamwork	Teamwork	Communication and teamwork
Leadership	Leadership	Task management	Task management

Improving social and cognitive skills in healthcare is critical but it is difficult to work towards becoming better if teams are not speaking the same teamwork language. At our simulation center, we found that teams from different clinical backgrounds bring with them different vernaculars related to these skills. This can lead to superficial discussions, lack of clarity and precision, and at worst misunderstanding and conflict. These subtle differences are rooted in historical differences and the rapid, at times diverging, application of team science in healthcare. A common language is the first step in getting teams “on the same page” in the quest for better teamwork.

Based on the problems experienced during multi-professional group training sketched in the vignette above, we have developed a generic tool, SCOPE, from existing approaches at the Copenhagen Academy for Medical Education and Simulation (CAMES). CAMES is a large, multi-professional simulation center with two locations. The location in which this study took place employed approximately 35 staff members, who provided the infrastructure, and approximately 110 healthcare professionals, who taught a variety of courses. Most of the approximately 12,000 participants per year are postgraduate professionals, working in different acute care settings.

SCOPE accommodates a shift in focus from analyzing the right or wrong of actions towards applying simulation for shared exploration, reflection, and learning across professions and disciplines and across a range of clinical situations [11, 12]. The tool is intended for educators, who design and conduct teaching sessions with and without simulation that aims to help learners understand and apply social and cognitive skills in their work context. It can also be used by clinicians who want to self-reflect about their work, or, together with others, debrief clinical cases. As researchers, we believe that we can contribute to understanding the dynamic complexity of the skills involved. In Table 3, we provide concrete ideas on how to use SCOPE.

For decades, various approaches have been used to optimize the role that human beings play in healthcare [1, 13–15]. They play a role in terms of concrete problem-solving in the here and now and in contributing to organizational development in the longer run and involving larger organizational units. This optimization can be interventive (e.g., by training) and/or analytic (e.g., by improving methods of needs analysis). Two different approaches to describing the role that humans play in healthcare have been especially prominent.

David Gaba and colleagues introduced ideas from aviation into healthcare under the label of crisis resource management, which evolved into crew resource management. Today this approach comprises 15 heuristic sentences that should help clinicians function well in the “complex and ill-structured real world” of clinical care [1, 16, 17].

Rhona Flin and colleagues introduced the term “non-technical skills” (NTS) as a heading that summarizes social skills such as teamwork, task management, and leadership as well as cognitive skills such as situation awareness and decision making [18–20]. Today there are a range of tools, so-called “behavioral marker systems” [7], available that were designed for different professions and specialties, for individuals and teams [8, 19, 21–26]. Several NTS tools were localized to, for example, the Danish context, including anaesthesiologists [27], anesthesia nurses [6], surgeons [4], and scrub nurses [5]. There are also approaches for developing new tools from scratch [28] and tools that were developed in different traditions [29].

We did consider the use of more generic frameworks, such as the principles of crew resource management [1, 30]. We decided to base our work on the NTS tools. First of all, we value the systematic build of the NTS tools with their set-up into categories and elements. Second, we also spent much time training our staff in the use of these tools. Finally, the tools are implemented in some of the official assessment processes for different professions in Denmark.

### The challenges in practice

Even though the tools look very similar, they differ at both the category and element levels. This is a natural consequence of their development process, involving clinicians and researchers who strove to make the tool reflect the social and cognitive skills needed in the respective clinical realities as best as possible. This resulted in tools that introduced a specific language to describe the social and cognitive skills in different settings. One might say that each tool represents a different dialect of an NTS language. While categories and elements can typically be understood and applied across professions and disciplines, the behavioral markers are typically target group-specific and not necessarily understood across disciplines.

The challenges described below are a result of informal observations within our internal faculty development program and reflective conversations. Three challenges became clear:

Whenever we were running multidisciplinary courses, scenarios, and debriefings were disturbed by the different dialects and the need to clarify the different terms. Facilitators either needed to spend some (often not planned) time during debriefing to sort things out, or they could, more or less, ignore the differences, contributing to the impression that the terms used are kind of arbitrary—“soft stuff”. This created obstacles in time management and/or educational alignment.

During debriefings, the use of the terms remained somewhat superficial. Facilitators and learners would say the “right” words but would often not go deeper than that. Words were said but would not always be related to a deep conceptual understanding. This again emphasized a certain feeling of arbitrariness and, to some extent frustration on the side of the facilitators.

As each tool is graphically designed as a table, there seemed to be a natural tendency to start with the category mentioned at the top and to make one’s way down during the debriefing discussion, which is not necessarily the best way to achieve the intended learning objectives. The sequence in the tool might not reflect the best sequence of discussion for a specific scenario. Given the complex nature of the events in a regular simulation scenario, there is always too little time for too many interesting discussions and fixed sequences might lead to spending time on issues that might not be the top priority for a scenario.

These challenges initiated an internal development project at CAMES. The aim of our project was to create a generic tool that should be easy to understand, that could be applied by different professions and disciplines and that could be presented graphically in a way to support users in building a more flexible concept of the terms used.

## The method and the results

We created a core working group with seven of our multi-professional course leaders and facilitators and a reference group consisting of 10 course leaders. All participants had several years of experience in simulation-based teaching that also included NTS elements. The core group did the main part of the development, involving the members of the reference group three times in the development process to obtain their feedback and to increase the likelihood of them being willing to implement it in their courses.

The first step in the process in the core group was to discuss existing NTS tools in English and Danish, for example, ANTS-dk [10], ANTS [26], NOTSS-dk [4], NOTSS [9], and SPLINTS-dk [5]. After the initial discussions, we agreed to work with the Danish versions only because we considered that they would overlap sufficiently with the English versions. The tools were printed and the elements of the different tools from the paper copies were cut out and sorted according to similarity in meaning. Some were identical across tools, and some were almost identical but different in wording. Over the course of four working meetings, we revisited the resulting tool in an iterative process. During the meetings we worked with the paper cut-outs and sorted them according to our understanding of semantic similarity, all the while discussing why we would place an element in which category. During those meetings, we shifted between working in the plenum and working in small groups of two to four participants. When differences in interpretations arose, and after referring to the literature that was the basis for the original tools, pedagogical concerns were often prioritized over theoretical/scientific precision. This process took approximately nine months. In the end, we agreed with version presented in Table 2.

**Table 2:**
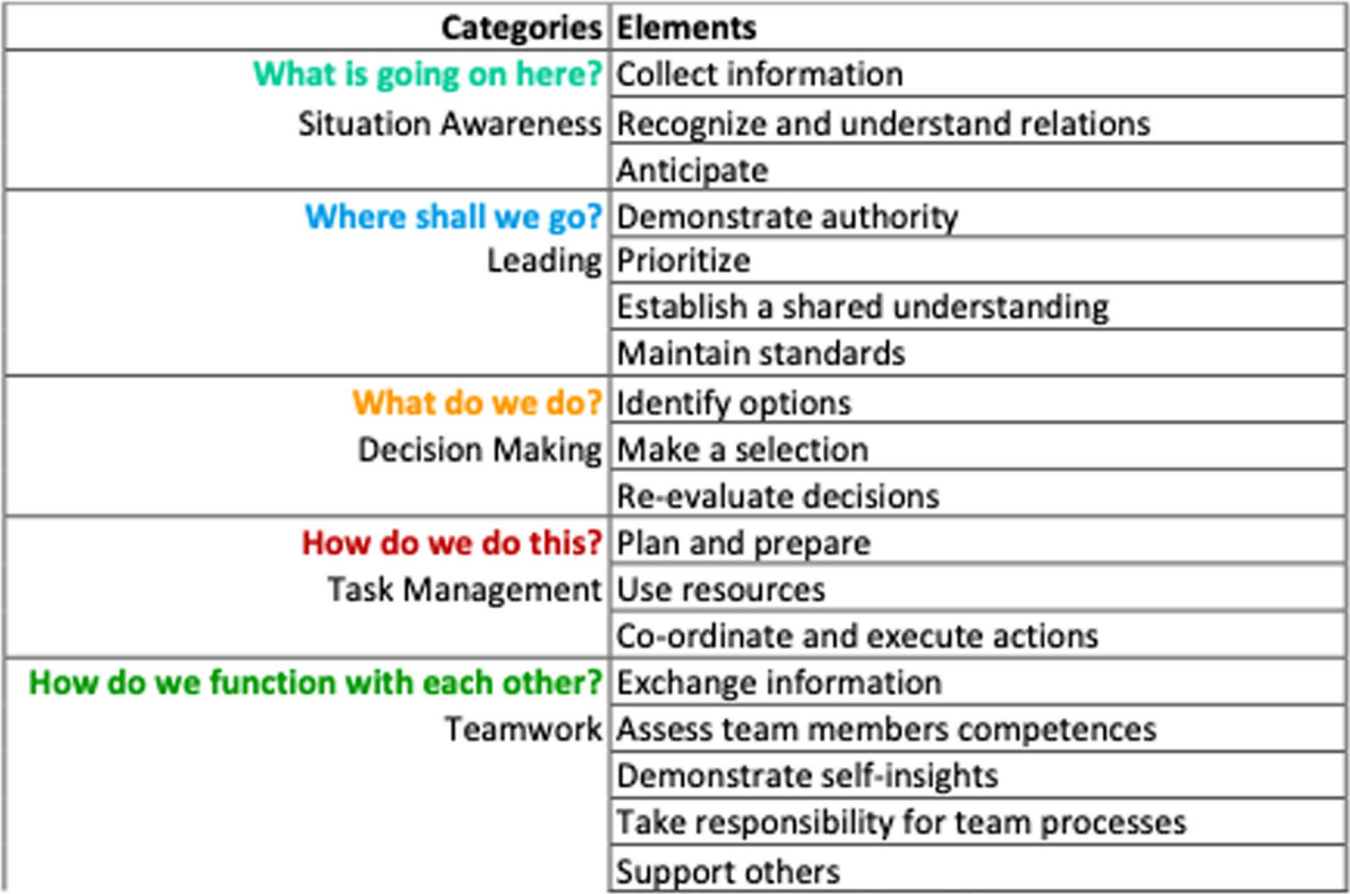
Overview of categories and elements in SCOPE

The social categories are “leading”, “teamwork”, and “task management”. The cognitive categories are “situation awareness” and “decision making”

We tried to boil down the essence of each category and element. We were inspired by Hannibal Lecter’s question to Clarice Starling in the movie “Silence of the Lambs”, asking her with reference to Marcus Aurelius: “For any particular thing ask: What is it in itself? What is its nature?” [31]. Even though concepts are always held by humans and therefore their nature will be interpersonally different and change over time, we thought this to be a valuable guiding idea. A helpful move in trying to capture the essence of categories was to formulate simple questions for each of them that evoked their “nature”. After repeated discussions, we developed the questions in Table 1. “Translating” the abstract terms from earlier tools into these straightforward questions also addressed the challenge mentioned above that the language associated with social and cognitive skills is often not part of the everyday terminology of most clinicians.

We discussed the need to represent the dynamic nature of how all the categories and elements involved may be relevant in any given and constantly changing situation. For example, when a healthcare team is in the process of diagnosing a patient, the cognitive elements may call for analytical attention. When implementing a decision about a treatment in a team the social elements in the model may be more relevant to reflect on. When looking closely at situations through these categories, we see how the ‘cognitive’ process of diagnosis may be highly affected by expressions of elements in the social category of leadership or teamwork. For example, when a hierarchy between healthcare professionals gets in the way of collecting information because one does not dare to ask the other. The unfolding of social elements, such as task management, may be affected by elements of the cognitive skills, if for example many tasks are carried out without reassessing decisions at intervals. In fact, the distinction between social and cognitive categories and elements is for analytical and educational purposes only. In practice, they are deeply intertwined [32–34]. SCOPE has inherited a distinction between social and cognitive skills. This distinction enriches analyses and discussions, but skills from different categories are often carried out simultaneously within the individual or within a team. Therefore, it seems infeasible in practice to, for example, talk about decision making without situational awareness, teamwork without task management, or situation awareness without considering other people involved into account. Our language, and the categories we apply to order our understanding, are limited and our concepts should not be taken for *truths* as such. This acknowledgement of SCOPE being a set of constructs or a very particular set of glasses through which to see situations unfold is something we also try to convey to the users of SCOPE. Any healthcare situation is so much richer than any one analytical framework can contain. However, we believe that SCOPE can help improve education and clinical work in healthcare.

Describing this dynamic relationship between the categories and elements themselves and the relationship between the categories and elements and the care situation was influenced by a workshop we ran at the International Meeting on Simulation in Healthcare (IMSH) and at the Annual Conference of the Society in Europe for Simulation Applied to Medicine (SESAM) in 2022. The workshop was inspired by organizational constellations [35], which we turned into “conceptual constellations”.

**Fig. 1:**
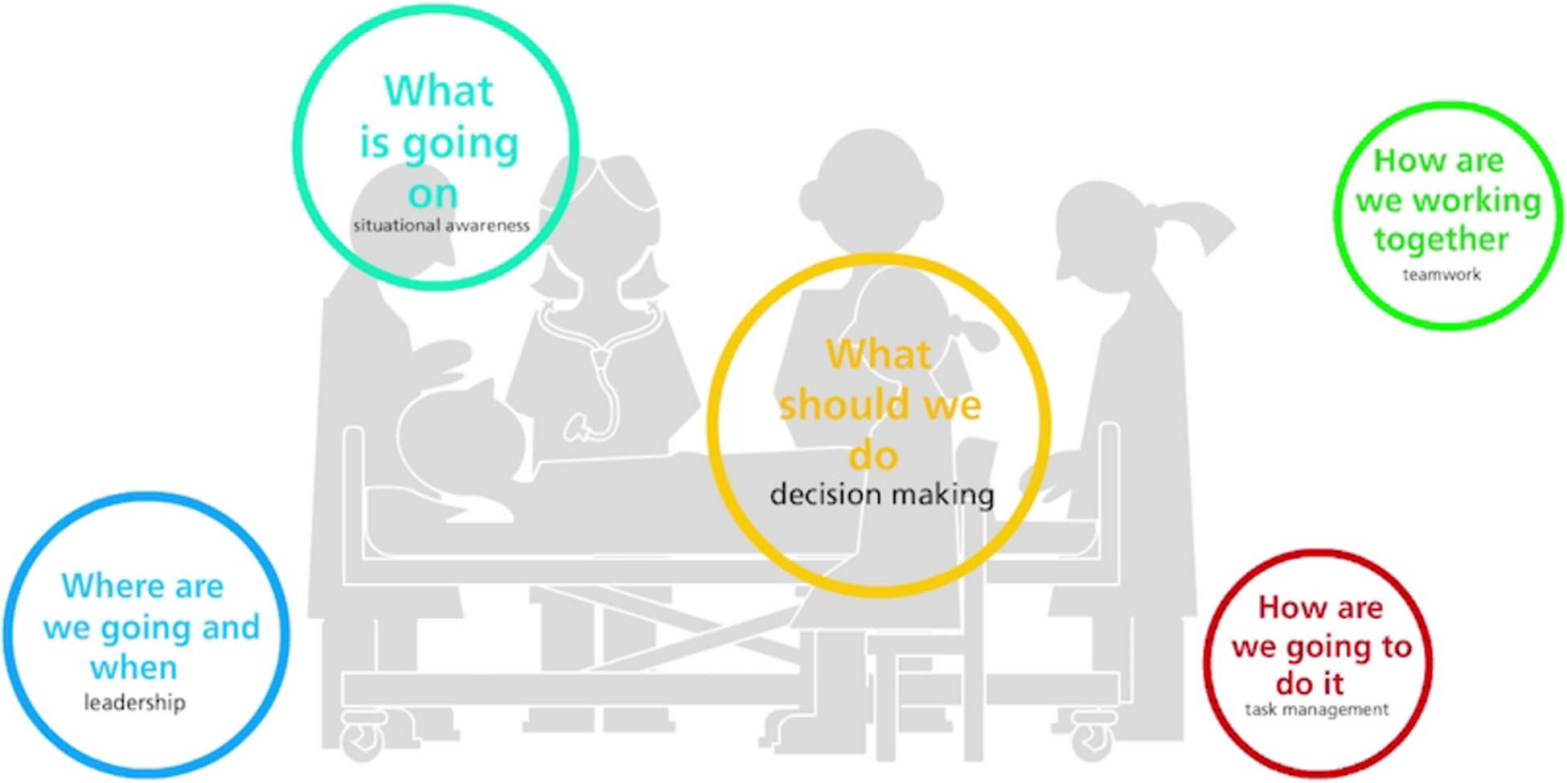
Starting constellation of the SCOPE animation. The full animation can be seen in the online appendix, and we recommend seeing it. The bubbles of the different categories have different sizes to illustrate that their relative importance in each situation can vary

The dynamic interplay of the elements is mirrored in an animated presentation of the SCOPE categories and guiding questions. As an alternative to presenting categories in a table, we developed an animation with categories changing size, moving from periphery to center, and merging with other categories to make the point that all situations may call for all categories, but their importance may change, and they may be difficult to separate analytically from each other. Figure 1 shows the starting point of the animation, and the full animation can be seen in the appendix (based on the feedback we received thus far, we highly recommend seeing it).

The name of the tool, SCOPE, describes the social (leading, task management, teamwork) and cognitive (situation awareness, decision making) skills (see Table 2). We do emphasize that, in our understanding such skills can be trained and improved and that they are not only a matter of talent, disposition, or personality.

This label, SCOPE, reflects our stance in the debate about terminology for the so-called “non-technical skills” [36, 37]. We find the term “non-technical skills” unhelpful, because it implies a dichotomous, and perhaps even hierarchical, distinction between a wide variety of skills and competencies that we know to be deeply entangled in safe practice [37].

## Discussion and reflection

We developed a generic tool to address social and cognitive skills in healthcare simulation and healthcare. SCOPE comprises five categories and 19 elements that are generic and can be used across professions, disciplines, care settings, and other contexts.

With regard to the challenge of working with several different tools on the same team, we acknowledge that there can be good reasons to make any tool very specific to professions and specialties. One important reason could be the need for a tool on which to base a formative or summative assessment of an individual´s social and cognitive skills. The process of developing such tools will be very valuable for the groups involved in the work—one needs to discuss and to some extent agree on how to read, interpret, and use the words contained. Those involved in these discussions will form certain concepts around the words, and thus reach a consensus of what they mean that can be shared by at least some for some time. This might be one of the good reasons for developing new tools, as for example, a recent project that started afresh to identify how NTS would be relevant for describing the competencies and competency levels of medical students [28]. Developing new and more specific tools, however, may also lead to the disadvantage of not being able to speak a common language across professions and education levels as discussed in the introduction. Murphy and colleagues emphasize how much words matter, and therefore the reflection about what we mean, when we develop constructs such as SCOPE is important [38]. Without knowing how far or not a consensus is reached across persons and time it is still important for the analysis of safe, effective, and efficient healthcare and the well-being of healthcare professionals as well as in healthcare education to have a shared framework or a tool to refer to. Words also have power and signify and transport power structures, which is another reason why we should reflect on how we use them [39].

In our view, the SCOPE categories and elements are sufficiently generic to be applied to basically any situation in which human beings interact with each other, with technologies, and with organizations. This would include clinical situations but also different types of simulation-based scenarios, including those that revolve around dilemmas and challenges for which there are no algorithmic approaches. Generic does not necessarily, however, mean nonspecific. Whereas categories and elements may be left unchanged, we strongly encourage teams to discuss markers and tailor them to specific situations and contexts that may involve several different professions or distributed teams. The process of discussing markers and perhaps formulating positive and negative examples will, we expect, heighten a shared awareness of what social and cognitive skills are for them in their specific working situations.

We have started applying the SCOPE tool in our simulation center when analyzing simulations and in our ongoing faculty development. The use of everyday questions has already received positive feedback from our simulation educators and course participants from the clinic. Currently, we do, however, not have any data about whether SCOPE and its setup actually help to address and understand the underlying concepts in more detail. This could be the focus of empirical investigations.

With regard to presenting the categories as an animation, we try to convey how the relevance and prominence of categories and elements as analytical lenses may shift. Categories and elements are lenses that can be useful for identifying certain aspects of a case at a certain point in time. They do not express “truths” but are tools to think, discuss, explain, to make sense of. There can be interpersonal consensus about how categories and elements apply to a case when used in relation to a case with specific questions in mind. In addition, this graphic representation of SCOPE dynamically captures the reality of how actual teaching and clinical situations unfold.

When we presented our new tool to our colleagues in different formats, one of the questions that we got early on was: where are the emotions? We very much agree that recognizing one’s own emotions and those of others makes it easier to manage them and to recognize that they influence our cognition and actions [32, 40–42]. However, with the resources that we had available, we could not accommodate integrating research about emotion, cognition, and (team)action into the SCOPE tool.

Indeed, the SCOPE tool does not explicitly include emotional competencies, just as it does not refer to the so-called technical abilities that are also always at play in simulations and working situations; rather, the fact that they are not explicitly mentioned does not prevent, of course, that they are drawn into individual and shared reflections on the same situations. At times the question “How did you feel in this situation?” might be enough. At times it might be valuable to supplement it with “What did trigger your emotions?” and/or “What were the effects of your emotions? On you? On the team? For the patient?”

We would like to acknowledge that “situational awareness” in particular was critiqued as a problematic construct, as it is almost always to be “assessed” only after the fact and therefore easily subject to counterfactual thinking and hindsight bias and might be used to judge people [43–49]. We kept it in this revision of SCOPE, as the term is established, and our process was not designed to address the conceptual foundation of the existing tools from which we combined SCOPE.

### Anticipated effects—towards a research agenda about SCOPE

When working with SCOPE, we anticipate that course participants and clinicians will use the concepts contained in a more harmonized way. We hope that some of the problems that we described in our starting vignette can be solved: it is only one poster to put up in the debriefing room, people will know where to look, and participants will (likely) know that leadership is contained in the framework for all. However, just having a taxonomy does not mean that people use it, interpret it in similar ways, or apply it to their learning and work in healthcare. Therefore, it will be important to develop effective ways to teach it in different contexts and to different people. The idea of bingo can be applied to help participants improve their understanding of the words, where they apply them to different movie snippets [50]. Another exercise, we developed is called “hand-it-on” which requires participants to actively apply SCOPE elements, while they stand in a circle and pass on different objects to each other according to specified rules [51].

We would expect similar effects when SCOPE is used in clinical practice. In addition, we anticipate positive effects on group dynamics between professions and disciplines as all involved would refer to the same tool. SCOPE’s set-up on the other hand will allow it to balance its generic value on the level of categories and elements with the possibility of discussing positive and negative behavioral markers. Our experience with the development of SCOPE showed us that these discussions have great value for the understanding of those involved. Additionally, seeing our SCOPE animation together seems to provide much intuitive sense of the dynamic nature of SCOPE.

For research, we hope to stimulate studies that investigate the concrete connotations that different people in different contexts form about the words contained. “Teamwork”, “task management”, etc., look different depending on who uses these words in what contexts. We argue that we need an improved understanding of those differences to understand where they make a difference [52]. We also hope that our animation can trigger new ways of thinking about how to assess SCOPE-related skills over time and in relation to other constructs.

### About the implementation

In our center, we have conducted several workshops with our course leaders about how to implement the new tool in the courses. The core group was available to discuss with course leaders how to implement SCOPE in existing scenarios and debriefings. A new model has several implications, as a substantial number of faculty members need to be retrained, and teaching materials need to be reworked. At this point, it is too early to assess the effects, but initial impressions are positive. SCOPE has been presented to clinicians, who appreciate that the tool is generic and can be applied in interdisciplinary teams. They also appreciate our reworking of the terminology from abstract labels of categories to every day guiding questions. Nevertheless, it remains a challenge to connect SCOPE terms to clinical tasks—this is where the real work is. We hope that we can stimulate you to work with SCOPE in different settings to explore its value and limitations, as well as the conditions under which it can play those out. Table 3 provides exemplary ideas on how to work with SCOPE in practice. We base these suggestions on the phases of simulation use. The phases could occur in a teaching, as well as in a research context. The aim and objectives would influence the concrete actions in the teaching context. The research question would provide guidance in a research setting.

**Table 3:** Ideas for working with SCOPE in and beyond simulation-based practices

Scenario design	Design scenarios in a way that they have the best chances to provide learning opportunities around SCOPE (e.g., distribute the qualifications that are needed to solve the case to different, instructed role players; place information that points to different differential diagnoses into the scenario) Ask role players in the scenario to significantly change their behavior and attitude following a certain event (e.g., the leader, who suddenly gets a blackout; the patient who outright rejects a treatment option now that was agreed upon before)
Scenario conduct	Adjust the challenges in the scenario so that there is more material for the debriefing (e.g., ask the simulated patient to ask for “other potential problems”, if the team is in danger of fixating on a wrong differential diagnosis). Stimulate (e.g., with a phone call) to use a SCOPE element that might be especially helpful in the current situation (e.g., is there anybody in your team, who could help with the challenge that you are dealing with?)
Debriefing	Analyze the “essence” of good solutions and problems during the scenario with the questions provided Analyze the dynamic changes over the course of the scenario for categories or elements, using timeline drawings or the concept constellations as described above. Analyse where categories and elements “merged” and then “separated” again—how and in what way did they influence each other? How long did that influence last?
Non-simulation teaching sessions	Provide the categories and elements to the learners and ask them to develop positive and negative behavioral markers for the elements from their own work context Use the idea of the dynamic SCOPE bubbles to work with a concept constellation as described above

Without having performed systematic studies, we believe that SCOPE contributed to alleviating some of the challenges that we described in the “challenges in practice” section. It seems that the words contained in SCOPE are used more and with a greater degree of understanding between different participants and educators.

### Where to from *here*?

We hope that SCOPE can help educators to design and implement even more effective teaching sessions. Based on the feedback in our presentations and the review process for this article, especially the animation can help learners obtain insights into the complex nature of social and cognitive skills that are difficult to generate otherwise.

Additionally, SCOPE might help clinicians reflect on their own practices and those of their colleagues. The focus points in the debriefing section can guide the use of the tool in this regard.

For the research community, we hope that we can address the dynamic nature and complexity of the issues that we are dealing with when talking about social and cognitive skills. Any summarizing measurement would need to find ways to somehow integrate the dynamic changes over time and would also need to account for the mutual influences of the concepts involved. Another question would be whether the social and cognitive skills should be treated on an individual or team-based level.

## Conclusion

We presented a generic tool for social (leading, task management, teamwork) and cognitive (situation awareness, decision making), SCOPE. SCOPE combines existing NTS tools, and is constructed to be applicable not only in different educational and healthcare contexts for individuals but also in mono- and multi-professional teams. SCOPE is organized around common-sense questions that should help users to more quickly form a conceptual understanding of the words used. An animated presentation of the categories in SCOPE emphasizes the dynamic nature of social and cognitive skills and how they may relate to each other and to educational and clinical situations.

### Supplementary Information

Supplementary Material 1.

## Data Availability

Not applicable.

## Abbreviations

ANTS: Anaesthesia non-technical skills system
ANTS-dk: Danish anesthesia non-technical system
CAMES: Copenhagen Academy for Medical Education and Simulation
IMSH: International Meeting on Simulation in Healthcare
NOTSS: Non-technical skills for surgeons
NOTSS-dk: Danish non-technical skills for surgeons
NTS: Non-technical skills
SCOPE: Social and cognitive skills taxonomy
SESAM: Society for Simulation in Europe
SPLINTS: Non-technical skills of the scrub practitioner
SPLINTS-dk: Non-technical skills of the scrub practitioner in Denmark
